# Revisiting the Intriguing Electronic Features of the BeOBeC Carbyne and Some Isomers: A Quantum‐Chemical Assessment

**DOI:** 10.1002/anie.202007990

**Published:** 2020-07-28

**Authors:** Jilai Li, Caiyun Geng, Thomas Weiske, Mingfei Zhou, Jun Li, Helmut Schwarz

**Affiliations:** ^1^ Institute of Theoretical Chemistry Jilin University 130023 Changchun China; ^2^ Institut für Chemie Technische Universität Berlin 10623 Berlin Germany; ^3^ Department of Chemistry Collaborative Innovation Center of Chemistry for Energy Materials Shanghai Key Laboratory of Molecular Catalysts and Innovative Materials Fudan University 200433 Shanghai China; ^4^ Department of Chemistry & Key Laboratory of Organic Optoelectronics and Molecular Engineering of Ministry of Education Tsinghua University 100084 Beijing China; ^5^ Department of Chemistry Southern University of Science and Technology 518055 Shenzhen China

**Keywords:** carbyne radicals, electronic configuration, quantum-chemical calculations, spin states

## Abstract

Extensive high‐level quantum‐chemical calculations reveal that the rod‐shaped molecule BeOBeC, which was recently generated in matrix experiments, exists in two nearly isoenergetic states, the ^5^Σ quintet (^5^ **6**) and the ^3^Σ triplet (^3^ **6**). Their IR features are hardly distinguishable at finite temperature. The major difference concerns the mode of spin coupling between the terminal beryllium and carbon atoms. Further, the ground‐state potential‐energy surface of the [2Be,C,O] system at 4 K is presented and differences between the photochemical and thermal behaviors are highlighted. Finally, a previously not considered, so far unknown *C*_2*v*_‐symmetric rhombus‐like four‐membered ring ^3^[Be(O)(C)Be] (^3^ **5**) is predicted to represent the global minimum on the potential‐energy surface.

## Graphical abstract

**One way or another**: The coexistence of two isoenergetic electromers of the novel BeOBeC molecule, possessing practically indistinguishable IR features, has been revealed by quantum‐chemical calculations. Further, the triplet state of a so far unknown *C*_2*v*_‐symmetric rhombus‐like four‐membered ring ^3^[Be(O)(C)Be] is predicted to represent the global minimum on the potential‐energy surface in the thermal reaction of Be_2_ with CO.

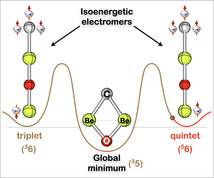

Molecules containing atomic carbon, for example transition‐metal carbides (TMCs), have been known since the end of the 19th century^1^ and have attracted quite some attention over the years, particularly in the field of industrial heterogeneous catalysis,^2^ for example in hydrodenitrogenation,^3^ hydrogenation,^4^ and Fischer–Tropsch synthesis.^5^ The interest is mainly due to the fact that some of these affordable TMCs, as for example WC,^6^ have similar properties to elements from the expensive precious platinum group and TMCs are regarded as novel materials for the capture, storage, and activation of CO_2_.^7^ Additionally, TMCs have been proposed as a new type of chemical tools to functionalize inert hydrocarbons.^8^ These investigations revealed a plethora of fascinating mechanistic scenarios for C−H bond activation,8c including a rare reaction‐induced localization of spin density in the thermal reaction of pristine [FeC_4_]^+^ with CH_4_.8b Or to give another example: The reactivity of diatomic [ReC]^+^ is without precedence in that the existence of long‐lived, isolated electronic states in a molecular system was demonstrated, and implications for the varying mechanisms of thermal dihydrogen splitting were uncovered.^9^ Demanding quantum‐chemical calculations proved necessary to properly describe the electronic structure of small metal carbides due to the multi‐reference character of many of these species.8c, 9, 10

Also of interest are systems which contain a monovalent carbon atom, the so‐called carbynes. The simplest carbyne, methylidyne (HC), was one of the first molecules to be identified as a constituent of interstellar space.^11^ Halocarbynes, for example, XC (X=F, Cl, Br) are known as short‐lived species,^12^ others serve as valuable intermediates in organic transformations,^13^ and transition‐metal complexes carrying a metal–carbon triple bond are well documented in the literature.^13^, ^14^

For simple carbyne radicals, both experiment and theory indicate that the reactivities of the doublet and quartet states of these molecules differ, and the electronic ground state most often corresponds to the doublet state;^15^ however, carbynes, having a quartet electronic ground state, were predicted to exist as well.^16^ In fact, quite recently, two of the present authors (M. Zhou and Jun Li) and their co‐workers reported the generation and characterization of a novel BeOBeC radical featuring an unusual electronic quintet ground state with a rare quartet carbyne unit comprising three unpaired electrons at the terminal carbon atom.^17^ This species was formed by irradiating co‐condensed beryllium and CO in a solid neon matrix. The initially produced Be_2_CO complex, upon exposure to visible light, rearranges to the insertion intermediate Be(CO)Be; the latter isomerizes further under UV irradiation to BeOBeC (Scheme 1). The products were characterized by IR spectroscopy, aided by using isotopic substitution. Based on quantum‐chemical calculations, for linear BeOBeC, a ^5^Σ state with an electronic configuration (α)BeOBeC(ααα), where the unpaired electrons at the terminal Be and C atoms are ferromagnetically coupled, was assigned as the ground state among all isomers considered. A triplet ^3^Π state with an (α)BeOBeC(αβα) electronic configuration, involving a doublet carbyne moiety, was calculated to be significantly less stable (>80 kJ mol^−1^) than the ^5^Σ state.^17^ Other electronic states, for example, a triplet with antiferromagnetic coupling of the unpaired electrons at the terminal Be and C atoms, that is, (β)BeOBeC(ααα), or alternative reaction pathways to convert Be_2_CO to BeOBeC and other conceivable isomers, were not reported.

**Scheme 1:**
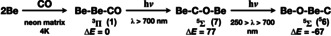
Generation of BeOBeC (^**5**^ **6**) according to ref. 17; energies, calculated using CCSD(T), are given in kJ mol^−1^ relative to BeBeCO (**1**).

Interest in carbyne‐related species and particularly in the often decisive role of spin states8b, 8c, 8f, 8g, 9, 10, 17, 18 has led us to further investigate the landscape of the [2Be,C,O] system at 4 K by advanced computational methods. We aimed at addressing the following questions:

1. Which multiplicity and which electronic configuration of BeOBeC actually correspond to the most stable electromer? Is the assignment of only one electronic state^17^ sufficient to describe the ground state of BeOBeC, namely the quintet ^5^Σ (^5^ **6**), or should the co‐existence of other spin states, for example, the triplet ^3^ **6** (^3^Σ), also be considered?
2. How does the potential‐energy surface (PES) for the thermal reactions of Be_2_ with CO look like? Are there other, perhaps more stable [2Be,C,O] isomers?

## Results and Discussion

### Interlude

A proper theoretical description of beryllium‐containing molecules is all but trivial. Even the beryllium dimer Be_2_—a seemingly simple molecule having only eight electrons—still poses an enormous challenge for its correct quantum‐chemical description. In fact, the Be_2_ case was considered “pathological”.^19^ As mentioned by Merritt et al., more than one hundred, often contradicting theoretical investigations on Be_2_ had already been published by 2009.19a Recently, in an extraordinarily demanding tour‐de‐force, the complete PES and the spectroscopic constants were reported for the ground state of X^1^Σ_g_^+^ of Be_2_,19b and this study emphasizes the need for both a proper theoretical method and adequate basis sets to cope with the often multireference character of these species.

### Electronic Configuration

A comparison of the experimentally obtained with the calculated IR spectrum of the linear BeOBeC isomer in its ^5^Σ state revealed a good agreement.^17^ The presence of a spin isomer, for instance the ^3^Σ species (^3^ **6**) in which the unpaired electron at the terminal beryllium atom is antiferromagnetically coupled to one of the unpaired electrons at the carbon unit, was not discussed in the previous publication.^17^

As shown in Table 1, extensive CCSD(T) calculations (for computational details, see the Supporting Information) reveal that both the triplet and quintet of BeOBeC (^**3,5**^ **6**) are almost isoenergetic at 4 K, with a tiny energetic preference for ^**5**^ **6**. We note that state‐specific CASSCF calculations indicate that ^**3**^ **6** features significant multireference character (see Figure S1).^20^ Thus, the CCSD(T) results may not be sufficiently reliable. Moreover, M06‐2X, widely used for Be‐containing systems,^21^ predicts even a reversed trend of stability, as does the ωB97 functional, which demonstrated excellent performance for MgO^+^ and Mg_2_O_2_^+^.^22^ Furthermore, out of 45 tested DFT functionals, 11 predict that **6** prefers a triplet ground state; for details, see Table S2.

**Table 1:** Enthalpy difference (in kJ mol^−1^) between ^3^[BeOBeC] (^**3**^ **6**) and ^5^[BeOBeC] (^**5**^ **6**) at 4 K as obtained using various methods with ^**5**^ **6** as reference.

method	Δ*H*
CCSD(T)/TZ	0.12
CCSD(T)/QZ	0.12
CCSD(T)/VTZ	0.13
CCSD(T)/VQZ	0.13
CCSD(T)/5Z//CCSD(T)/QZ	0.12
CCSD(T)/CBS//CCSD(T)/QZ	0.16
M06‐2X/AVQZ//M06‐2X/AVTZ	−0.29
ωB97/AVQZ//ωB97/AVTZ	−0.04

Since the IR spectroscopic features of ^**3**^ **6** and ^**5**^ **6** are practically indistinguishable (see Tables S3 and S4), an experimental differentiation and unambiguous assignment of these ferro‐ and antiferromagnetically coupled states would require an approach based on the different magnetic properties of the two multiplicities. In the absence of these data, a definitive assignment on the actual electronic configurations and spin states of BeOBeC (**6**), generated according to Scheme 1, is not possible.

**Figure 1:**
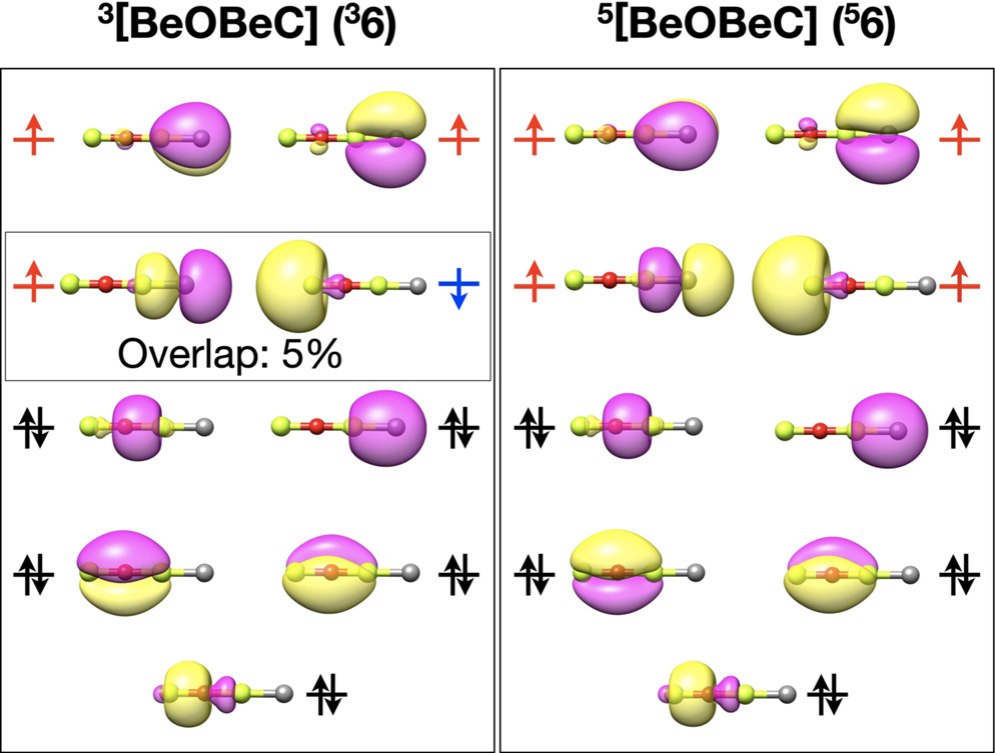
The occupied frontier orbitals of ^**3,5**^ **6** as obtained using B3LYP/aug‐cc‐pVTZ. Note that the natural orbital occupation as obtained using CASSCF are given in Figure S1.

Quite interestingly, a more detailed inspection of the spin density of the individual atoms of ^**3,5**^ **6** points to another unexpected facet. The spin distributions of ^**3,5**^ **6**, given in Table 2 and calculated by the Mulliken method, demonstrate that the antiferromagnetic coupling of the unpaired electron at the terminal beryllium through bonds reduces the spin density at the carbon atom. Moreover, for both spin states of **6** we note quite some delocalization of the spin as illustrated in Figure 1. Further, we note that the NBO‐based charge distributions of ^**3,5**^ **6** calculated at the CASSCF level are the same.

**Table 2:** Spin densities and charge distributions at each atom of ^**3**^ **6** and ^**5**^ **6** as obtained using CASSCF(14e,16o)/aug‐cc‐pVTZ.

	Be1	O	Be2	C
	Mulliken spin densities			
^3^[Be1OBe2C] (^3^ **6**)	−0.51	−0.05	0.23	2.33
^5^[Be1OBe2C] (^5^ **6**)	0.95	−0.02	0.27	2.79
	
	NBO charges			
^3^[Be1OBe2C] (^3^ **6**)	0.94	−1.81	1.56	−0.69
^5^[Be1OBe2C] (^5^ **6**)	0.93	−1.81	1.56	−0.69

### Potential Energy Surface

The BeOBeC isomer (**6**) was generated at 4 K by light‐induced isomerization of Be_2_CO (Scheme 1).^17^ Mechanistic details of this reaction, which proceeds via excited electronic states of Be_2_CO, are not known. The same holds true for the so far unexplored PES at 4 K of the [2Be,C,O] system; this aspect will be addressed briefly next.

**Figure 2:**
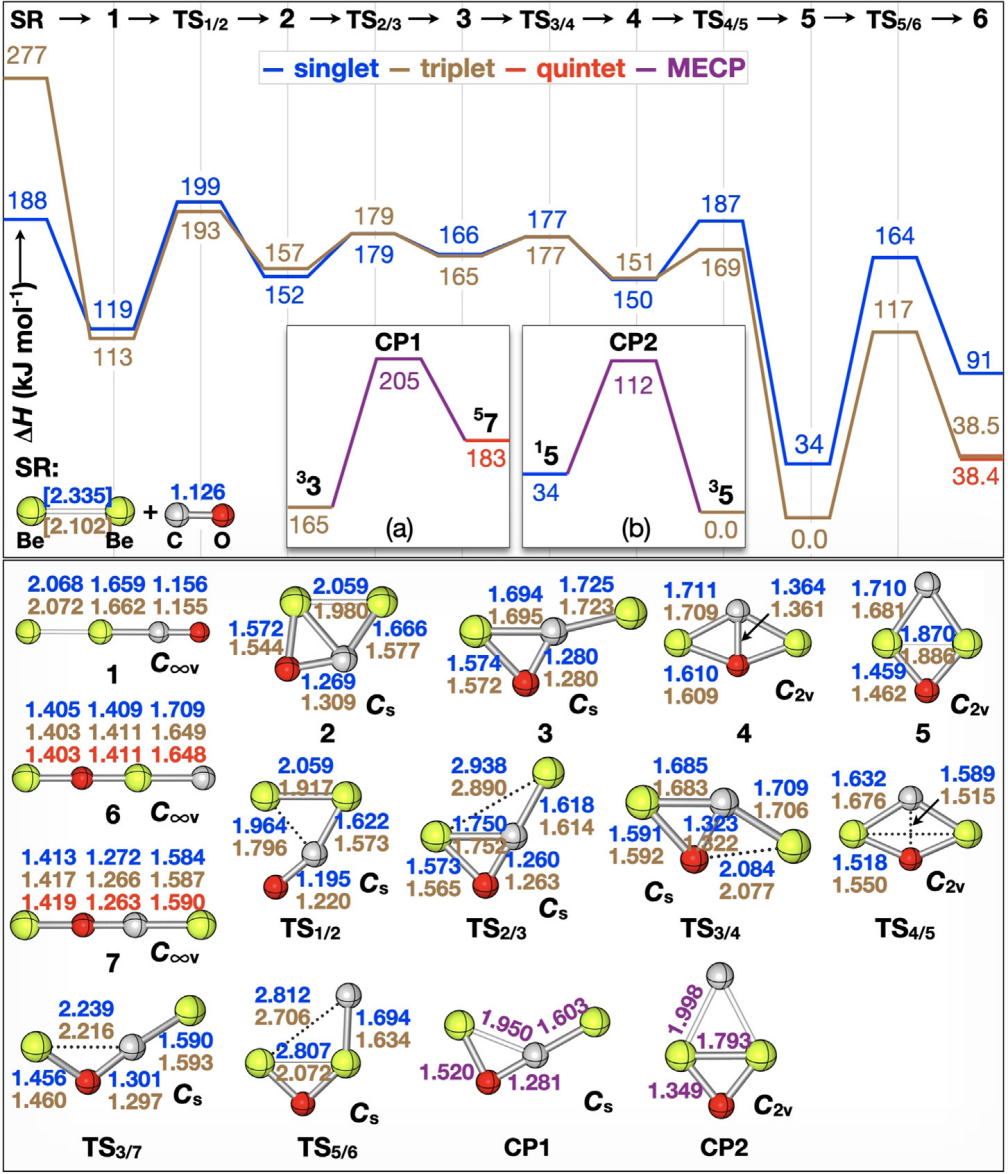
Simplified PES (Δ*H*_4 K_ in kJ mol^−1^) and optimized structures (bond lengths in Å) for the reactions of Be_2_ with CO as obtained using CCSD(T)/CBS//B3LYP/aug‐cc‐pVTZ. The insets display the minimum‐energy crossing points (MECPs). Color codes: singlet: blue; triplet: brown; quintet: red, MECP: purple. Note that the energy of ^1^Be_2_+CO was derived by calculating the relative energy of ^1^Be_2_ and ^3^Be_2_ using CASPT2(4e,8o)/aug‐cc‐pVQZ due to the multireference character of the beryllium dimer (ref. 19b). Bond lengths of Be_2_ in brackets are obtained using CASPT2/aug‐cc‐pVQZ. For the PES, based on B3LYP energetics, see Figure S2.

As shown in Figure 2, there is a smooth conversion when the beryllium dimer is reacted thermally with CO. At the singlet ground state, the encounter complex **1** is formed in a barrier‐free process. While in the condensed‐phase, for example, in a neon matrix, the energy gained in generating **1** is dissipated to the environment and intermediate **1** is trapped in the potential well, in the gas phase this energy is not liberated but stored as ro‐vibrational energy in **1**. Thus, under collision‐free conditions, “hot” **1** may continue to react, and the rate‐limiting step to the formation of the various products is associated with **TS_1/2_**. As the latter is located only 11 kJ mol^−1^ above the separated reactants, the internal energy of Be_2_/CO may already be sufficient to overcome **TS_1/2_** without additional energy input. All remaining transition states and intermediates along the reaction coordinate to eventually generate **6** are located below or close to the entrance channel. Also, the minimum‐energy crossing points (MECPs)^23^ at which changes of the multiplicities may occur, are energetically accessible. We note that ^**3,5**^ **6**, in addition to being almost isoenergetic, exhibit both practically identical geometric features (Figure 2) and NBO‐based charges (see Table 2). This explains why their IR spectroscopic features are hardly distinguishable (Tables S3 and S4). Similarly, at the triplet surface, all species considered proceed through submerged intermediates/transition states relative to the entrance channel of ^3^Be_2_/CO. Interestingly, the global minimum in the [2Be,C,O] system does not correspond to the linear BeOBeC species **6** as mentioned earlier;^17^ rather, it is the triplet of the *C*_2*v*_‐symmetric rhombus‐like four‐membered ring ^**3**^ **5** which is significantly more stable than **6** (see, Tables S5 and S6). Obviously, under thermal conditions the generation of ^**3**^ **5** would clearly dominate the whole scenario. ^**3**^ **5** is predicted to have six vibrational fundamentals, all have appreciable IR intensities (see, Figure S3 as well as Tables S3 and S4). However, there is no experimental evidence in forming this isomer in the light‐induced reaction of Be_2_ with CO in solid neon.^17^, ^24^, ^25^ While the IR absorption band at around 1150 cm^−1^ could appear to be assigned to **5** (Tables S3 and S4), the isotopic shifts obtained by ^18^O and ^13^C labelling rule out this assignment; rather, they are only compatible with linear Be(CO)Be (**7**).^17^, ^24^

## Conclusion

In conclusion, in this computational study, novel insight is provided into the features of some [2Be,C,O] isomers. Quite importantly, for the IR‐spectroscopically identified isomer BeOBeC (**6**), the actual multiplicity has yet to be determined, as both the almost isoenergetic quintet and triplet states of **6** cannot be distinguished unambiguously by the currently available methods. Most likely, these two differently spin‐coupled electronic states co‐exist at finite temperature. Not surprisingly, the pathways in the thermochemical and photochemical transformations of [2Be,C,O] differ, although some of the products, for example, **6** and **7**, are accessible on either route. While the global minimum Be(O)(C)Be (**5**) cannot be formed photochemically,^17^ it would require only a small thermal activation of the Be_2_/CO couple to create this hitherto unknown rhombus‐like four‐membered ring.^26^

## Conflict of interest

The authors declare no conflict of interest.

## Supporting information

As a service to our authors and readers, this journal provides supporting information supplied by the authors. Such materials are peer reviewed and may be re‐organized for online delivery, but are not copy‐edited or typeset. Technical support issues arising from supporting information (other than missing files) should be addressed to the authors.

Supplementary
