# Supplementary material for: Revisiting the Intriguing Electronic Features of the BeOBeC Carbyne and Some Isomers: A Quantum‐Chemical Assessment
Source: Angew Chem Int Ed Engl. 2020 Jul 28;59(39):17261–5. doi: 10.1002/anie.202007990 (PMC7540417; doi:10.1002/anie.202007990)
Supplement: Supplementary file 1 — Supplementary [file ANIE-59-17261-s001.pdf]

Angewandte Chemie
Eine Zeitschrift der Gesellschaft Deutscher Chemiker

Supporting Information

Revisiting the Intriguing Electronic Features of the BeOBeC Carbyne and Some Isomers: A Quantum-Chemical Assessment

Jilai Li, Caiyun Geng, Thomas Weiske, Mingfei Zhou,* Jun Li,* and Helmut Schwarz**

anie_202007990_sm_miscellaneous_information.pdf

Table of Contents

1. Computational Details 2
2. Figures 3
3. Tables 6
4. Coordinates of key isomers of [2Be,C,O] 11
5. References 13

1. Computational Details

The calculations of the electronic structures were performed with ORCA, Gaussian and MRCC.[1]

We used the B3LYP[2] density functional in combination with the aug-cc-pVTZ[3] basis set for the structural optimization to model the potential energy surfaces of the thermal reactions. Harmonic vibrational frequencies were computed to verify the nature of the stationary points. The minimum structures reported in this paper show only positive eigenvalues of the Hessian matrix, whereas the transition states (TSs) have only one negative eigenvalue. Intrinsic reaction coordinate[4] calculations were also performed to confirm that the transition states correlate with designated intermediates. The thermodynamic functions (ΔH) were estimated within the ideal gas, rigid-rotor, and harmonic oscillator approximations at 4K and 1 atm.

As commonly accepted, the geometries of molecular structures are less dependent on the level of theory than the energies. Therefore, for further energetic refinements, single-point energy calculations by using the CCSD(T) method in combination with various basis sets such as aug-cc-pVTZ (AVTZ), aug-cc-pVQZ (AVQZ), and ANO-pVTZ:ANO-pVQZ[5] extrapolated complete basis set (CBS)[5-6] were performed for the structures as optimized by the aid of the B3LYP functional. The energetic information is given in Table S1.

Quite elaborate multireference (MR) calculations were conducted to determine the relative energies of low-lying electronic states of Be_2. To treat dynamic correlation without the problems of intruder states or level shifts,[7] CASPT2[8] in conjunction with the aug-cc-pVQZ basis set, as implemented in ORCA, was employed to optimize the geometries of Be_2; the active space (4e,8o) with 4-electron in 8 orbitals has been considered in these calculations.

For the calculations of the different spin states of ^{3,5}**6**, the state-specific complete active space self-consistent field (CASSCF)[9] approach in conjunction with the aug-cc-pVTZ basis set was conducted to obtain the electronic configuration. An active space (14e,16o) has been considered; for the selection of the active space, see Figure S1. In addition, we also optimized the structures by using the CCSD(T) method with various basis sets such as aug-cc-pVTZ (AVTZ), aug-cc-pVQZ (AVQZ), cc-pVTZ (VTZ), aug-cc-pVQZ (VQZ), and refined the energy by using aug-cc-pV5Z (AV5Z) and CBS basis sets, respectively. The energetic information is given in Table 1.

In addition, single-point energy, vibrational frequencies (cm^{-1}), IR intensities (km mol^{-1}) of selected isomers by various DFT and MP2 methods in conjunction with the aug-cc-pVTZ basis set were also performed; the data is given in Tables S2, S3 and S5.

2. Figures

Figure S1. The selected active spaces considered in the CASSCF(14e,16o)/aug-cc-pVTZ calculations for the triplet and quintet states of BeOBeC. Natural orbital partial occupation numbers and roots are given.

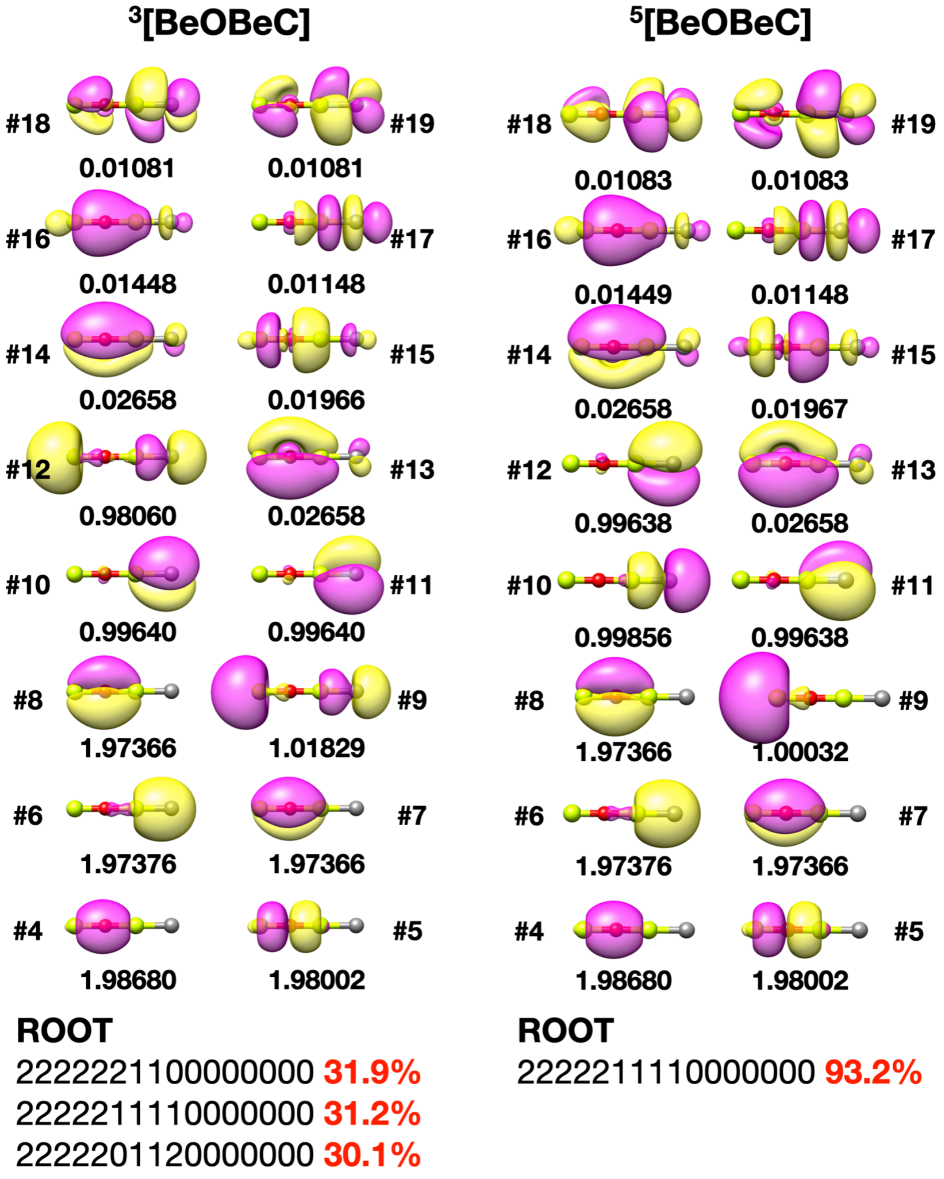

Figure S2. Simplified PES (ΔH_{4K} in kJ mol^{-1}) and optimized structures (bond lengths in Å) for the reactions of Be_2 with CO as obtained at the B3LYP/aug-cc-pVTZ level of theory. The insets display the MECPs. Color codes: singlet: blue; triplet: brown; quintet: red, MECP: purple.

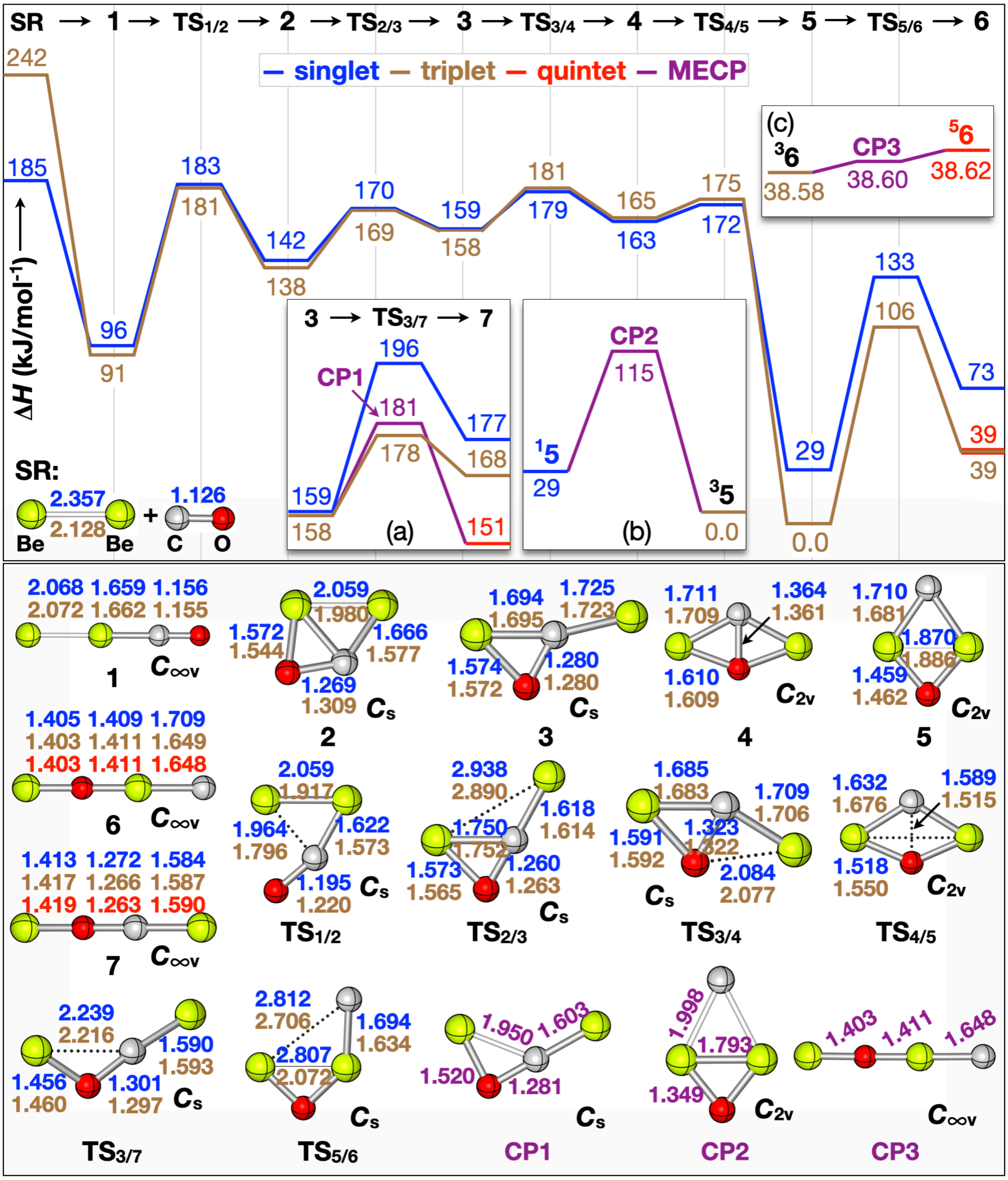

Figure S3. IR spectra of [3]**5** and the assignment of vibrational modes as obtained at the B3LYP/aug-cc-pVTZ level of theory.

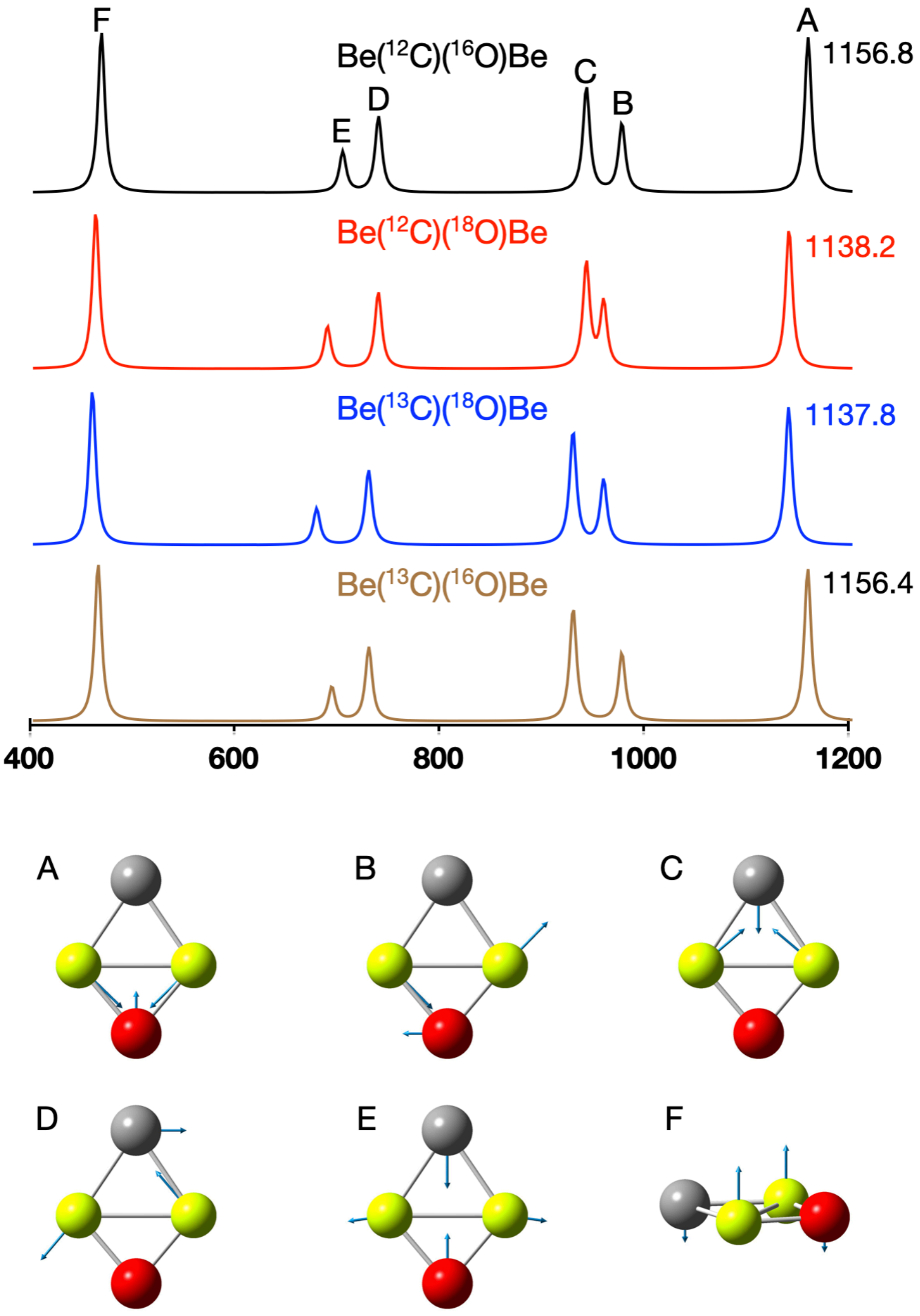

3. Tables

Table S1. Energetics (in kJ mol^{-1}) as obtained at the B3LYP/aug-cc-pVTZ level by using Gaussian and ORCA and at the CCSD(T)/aug-cc-pVTZ (AVTZ), CCSD(T)/aug-cc-pVQZ (AVQZ), CCSD(T)/ANO-cc-pVTZ:ANO-cc-pVQZ (CBS) levels by ORCA. $\Delta\Delta H$ gives the errors of B3LYP as compared with CCSD(T)/CBS. The T1 diagnostic values of the CCSD(T) calculations are provided as well. Numbers given in red suggest that the data based on the CCSD(T) approaches should be viewed with caution.

	B3LYP		CCSD(T)				
	Gaussian	ORCA	AVTZ	AVQZ	CBS	$\Delta\Delta H$	T1
**$^1Be_2$+CO**	185	186	172	198	199	13	0.094
**$^3Be_2$+CO**	242	242	245	274	277	35	0.014
**^{1}1**	96	96	106	119	119	23	0.020
**^{3}1**	91	91	100	113	113	22	0.020
**^{1}TS1/2**	183	183	198	199	199	16	0.053
**^{3}TS1/2**	181	181	200	193	193	12	0.064
**^{1}2**	142	142	156	152	152	11	0.057
**^{3}2**	138	138	165	157	157	19	0.085
**^{1}TS2/3**	170	170	179	179	179	9	0.074
**^{3}TS2/3**	169	169	180	179	179	10	0.073
**^{1}3**	159	159	165	166	166	6	0.020
**^{3}3**	158	158	164	165	165	6	0.036
**^{1}TS3/4**	179	179	177	177	177	-3	0.020
**^{3}TS3/4**	181	180	179	177	177	-3	0.019
**^{1}4**	163	163	159	150	150	-13	0.019
**^{3}4**	165	164	161	151	151	-13	0.018
**^{1}TS4/5***	172	172	201	187	187	15	0.071
**^{3}TS4/5**	175	175	180	169	169	-6	0.040
**^{1}5**	29	29	34	34	34	5	0.022
**^{3}5**	0	0	0	0	0	0	0.024
**^{1}TS5/6**	133	133	146	164	164	31	0.031
**^{3}TS5/6**	106	106	101	117	117	11	0.027
**^{1}6**	73	73	76	91	91	19	0.015
**^{3}6**	39	39	24	39	38.51	0	0.017
**^{5}6**	39	39	24	38	38.36	0	0.016
**^{1}TS3/7**	196	195	220	229	229	33	0.022
**^{3}TS3/7**	178	178	195	203	203	25	0.020
**^{1}7**	177	177	206	217	217	40	0.021
**^{3}7**	168	168	198	208	208	40	0.021
**^{5}7**	151	151	174	183	183	32	0.020
**CP1**	181	181	201	205	205	23	0.059
**CP2**	115	115	110	112	112	-3	0.045
**CP3**	39	39	24	39	39	0	0.017

Table S2. Energy differences (in kJ mol^{-1} without zero-point energy corrections) between ^{3}**6** and ^{5}**6** relative to the latter. Note, the AVTZ basis set was used in these single point energy calculations by using the geometries optimized at the B3LYP/AVTZ level.

	^{3}**6**
VWN	-0.04
VWN3	0.02
PWLDA	-0.03
BP86	0.10
BLYP	-0.45
OLYP	-0.22
XLYP	-0.57
PW91	0.14
mPWPW	0.21
mPWLYP	-0.62
PBE	0.14
RPBE	0.05
REVPBE	0.13
PWP	-0.09
B1LYP	-0.01
B3LYP	0.01
O3LYP	0.04
X3LYP	0.00
B1P	0.15
B3P	0.15
B3PW	0.21
PW1PW	0.17
mPW1PW	0.19
mPW1LYP	-0.03
PBE0	0.18
BHandHLYP	0.01
TPSS	0.08
TPSSH	0.11
TPSS0	0.10
M06	0.05
M062X	-0.58
B97M-D3BJ	0.22
wB97	-0.01
wB97X	0.06
wB97X-D3BJ	0.09
CAM-B3LYP	0.07
LC-BLYP	0.12
B2PLYP	0.09
mPW2PLYP	0.07
B2GP-PLYP	0.10
B2K-PLYP	0.12
B2T-PLYP	0.10
DSD-BLYP	0.13
DSD-PBEP86	0.16
wB2PLYP	0.12
wB2GP-PLYP	0.13
Average	0.03

Table S3. Calculated vibrational frequencies (cm^{-1}) and IR intensities ($km\ mol^{-1}$) of [2Be,C,O] isomers at the B3LYP/aug-cc-pVTZ level of theory.

	ν_1(cm^{-1})	I ($km\ mol^{-1}$)	ν_2(cm^{-1})	I ($km\ mol^{-1}$)
CO	2207.3	80		
^{1}**1**	2028.9	773		
^{3}**1**	2030.6	835		
^{1}**2**	1396.8	450		
^{3}**2**	1370.6	303		
^{1}**3**	926.9	205	1461.1	43.4
^{3}**3**	923.6	212	1460.8	38.6
^{1}**4**	998.8	224	1123.9	19.6
^{3}**4**	1003.9	252	1139.0	21.8
^{1}**5**	1156.0	120		
^{3}**5**	1157.1	110		
^{1}**6**	1155.4	0.4	1485.5	792
^{3}**6**	1190.1	0.7	1495.5	890
^{5}**6**	1190.3	0.1	1497.1	919
^{1}**7**	1170.5	458	1678.8	253
^{3}**7**	1159.4	415	1666.7	87.2
^{5}**7**	1150.9	412	1662.8	147

Table S4. Calculated vibrational frequencies (cm^{-1}), IR intensities ($km\ mol^{-1}$) of selected isomers by various methods in conjunction with the aug-cc-pVTZ basis set.

	^{3}**1**		^{3}**6**		^{3}**6**		^{5}**6**		^{5}**6**		^{5}**7**		^{5}**7**		^{3}**5**	
	vib	int	vib1	int	vib2	int	vib1	int	vib2	int	vib1	int	vib2	int	vib	int
**B3LYP**	**2031**	**835**	**1495**	**890**	**1190**	**1**	**1497**	**919**	**1190**	**0**	**1663**	**147**	**1151**	**412**	**1157**	**110**
BP86			1453	686	1158	1	1458	852	1158	0	1613	47	1100	337	1112	77
TPSS			1468	796	1169	0	1470	863	1169	0	1610	89	1116	357	1124	92
TPSSH	2011	826	1486	873	1183	0	1489	893	1186	0	1640	139	1136	395	1145	108
BH&HLYP	2115	1028	1541	972	1228	1	1542	976	1228	1	1732	316	1201	488	1212	160
BMK	2122	910	1546	971	1234	0	1547	973	1235	0	1456	689	971	37	1240	152
CAM-B3LYP	2067	835	1513	948	1208	0	1513	953	1208	0	1704	261	1179	468	1184	142
M062X	2088	896	1517	966	1210	1	1517	990	1210	1	1692	277	1175	482	1196	147
M06L	2035	1757					1511	917	1210	0					1176	122
M11	2069	783	1466	954	1162	0	1466	955	1162	0	1680	332	1129	524	1163	165
M11L			1471	727	1168	8	1476	935	1170	0	1695	118	1100	405	1155	118
PBE1PBE	2066	889	1508	921	1202	0	1508	925	1202	0	1692	175	1160	428	1171	122
X3LYP	2036	845	1499	892	1193	1	1500	920	1194	0	1668	158	1155	416	1160	112
wB97	2058	777	1474	961	1176	1	1475	963	1176	1	1698	345	1144	561	1161	169
B2PLYP	1984	775	1494	923	1187	0	1496	926	1189	0	1670	240	1155	450	1148	115
B2GP-PLYP	1999	816	1503	936	1195	0	1503	938	1195	0	1687	292	1164	471	1161	130
MP2	1957	836	1479	942	1176	0	1479	940	1176	0	1680	416	1148	508	1141	183

Table S5. Relative energies (in kJ mol^{-1}) of isomers **5** and **6** at 4K as obtained at the CCSD(T)/CBS(ANO-pVTZ:ANO-pVQZ)//B3LYP/aug-cc-pVTZ level of theory.

CCSD(T)	ΔH	ΔE
^{1}**5**	38	35.8
^{3}**5**	0.0	0.0
^{1}**6**	82	87.0
^{3}**6**	40	43.3
^{5}**6**	40	43.1

Table S6. Relative energies (in kJ mol^{-1}) for selected isomers as obtained by various methods in conjunction with the aug-cc-pVTZ basis set.

	^{3}**1**	^{1}**5**	^{3}**5**	^{3}**6**	^{5}**6**	^{5}**7**
**B3LYP**	**96**	**31**	**0**	**43**	**43**	**154**
BP86		31	0	67	67	144
TPSS		36	0	57	56	155
TPSSH	91	38	0	48	48	154
BH&HLYP	102	33	0	17	17	151
BMK	118	45	0	48	48	160
CAM-B3LYP	101	33	0	37	37	155
M062X	121	54	0	41	42	176
M06L	128	54	0		51	
M11	85	42	0	34	33	158
M11L		42	0	46	46	171
PBE1PBE	91	37	0	44	43	139
X3LYP	97	31	0	44	44	155
wB97	95	37	0	53	53	190
B2PLYP	98	36	0	39	39	165
B2GP-PLYP	101	39	0	32	32	164
MP2	104	72	0	22	23	167
CCSDT			0	28	28	

4. Coordinates of key isomers of [2Be,C,O] as obtained at the B3LYP/AVTZ level of theory

^{1}1

Be	0.000000	0.000000	2.669266
Be	0.000000	0.000000	0.601658
C	0.000000	0.000000	-1.057542
O	0.000000	0.000000	-2.213382

^{3}1

Be	0.000000	0.000000	0.000053
Be	0.000000	0.000000	2.072110
C	0.000000	0.000000	3.734123
O	0.000000	0.000000	4.888714

^{1}2

Be	-0.307534	1.375789	-0.469048
Be	-0.716676	-0.234509	0.747356
C	0.490252	-0.084298	-0.545904
O	0.533958	-1.056982	0.267596

^{3}2

Be	-1.216498	10.331511	-10.196017
Be	-2.472730	9.549852	-8.880513
C	-1.259964	10.483217	-8.498108
O	-0.329871	11.094461	-9.187565

^{1}3

C	0.098802	0.019165	0.369641
Be	-0.723015	0.021133	1.886690
Be	1.217935	0.044921	-0.901330
O	-0.351547	-0.051898	-0.826025

^{3}3

C	0.093548	0.018841	0.368857
Be	-0.720532	0.020528	1.887939
Be	1.218521	0.045431	-0.898268
O	-0.349362	-0.051478	-0.829552

^{1}4

C	-0.628109	0.000678	-0.461342
Be	-0.873291	-0.000316	1.231892
Be	0.914243	-0.000304	-1.201820
O	0.471023	-0.000035	0.345958

^{3}4

C	-0.626838	0.000470	-0.460409
Be	-0.872921	-0.000064	1.230784

Be	0.913297	-0.000052	-1.201136
O	0.470328	-0.000332	0.345449

^{1}5

Be	0.000000	0.376342	0.859315
Be	0.000000	-0.228593	-0.909887
C	0.000000	-1.281029	0.438038
O	0.000000	1.133280	-0.387466

^{3}5

C	0.000000	0.000000	0.000000
Be	0.000000	0.000000	1.681462
Be	1.561445	0.000000	0.623862
O	1.406943	0.000000	2.077216

^{1}6

C	-2.267351	0.004630	0.000000
Be	2.255255	0.018415	0.000011
Be	-0.557950	-0.016301	0.000013
O	0.851193	-0.003382	-0.000003

^{3}6

Be	0.000000	0.000000	-0.000130
O	0.000000	0.000000	1.402398
Be	0.000000	0.000000	2.813987
C	0.000000	0.000000	4.462744

^{5}6

Be	0.000000	0.000000	0.000000
O	0.000000	0.000000	1.403000
Be	0.000000	0.000000	2.814000
C	0.000000	0.000000	4.462000

^{1}7

Be	0.000000	0.000000	1.677739
Be	0.000000	0.000000	-2.592112
C	0.000000	0.000000	-1.007682
O	0.000000	0.000000	0.264410

^{3}7

Be	0.000000	0.000000	1.677865
Be	0.000000	0.000000	-2.591669
C	0.000000	0.000000	-1.004757
O	0.000000	0.000000	0.260916

^{5}7

Be	0.000000	0.000000	0.000000
C	0.000000	0.000000	1.590000
O	0.000000	0.000000	2.853000
Be	0.000000	0.000000	4.272000

5. References

[1] (a) M. J. Frisch, G. W. Trucks, H. B. Schlegel, G. E. Scuseria, M. A. Robb, J. R. Cheeseman, G. Scalmani, V. Barone, B. Mennucci, G. A. Petersson, H. Nakatsuji, M. Caricato, X. Li, H. P. Hratchian, A. F. Izmaylov, J. Bloino, G. Zheng, J. L. Sonnenberg, M. Hada, M. Ehara, K. Toyota, R. Fukuda, J. Hasegawa, M. Ishida, T. Nakajima, Y. Honda, O. Kitao, H. Nakai, T. Vreven, J. J. A. Montgomery, J. E. Peralta, F. Ogliaro, M. Bearpark, J. J. Heyd, E. Brothers, K. N. Kudin, V. N. Staroverov, T. Keith, R. Kobayashi, J. Normand, K. Raghavachari, A. Rendell, J. C. Burant, S. S. Iyengar, J. Tomasi, M. Cossi, N. Rega, J. M. Millam, M. Klene, J. E. Knox, J. B. Cross, V. Bakken, C. Adamo, J. Jaramillo, R. Gomperts, R. E. Stratmann, O. Yazyev, A. J. Austin, R. Cammi, C. Pomelli, J. W. Ochterski, R. L. Martin, K. Morokuma, V. G. Zakrzewski, G. A. Voth, P. Salvador, J. J. Dannenberg, S. Dapprich, A. D. Daniels, O. Farkas, J. B. Foresman, J. V. Ortiz, J. Cioslowski, D. J. Fox, Gaussian 09, Revision D.01, Gaussian, Inc., Wallingford CT, **2013**; (b) F. Neese, *WIREs Comput. Mol. Sci.* **2018**, *8*, e1327; (c) M. Kállay, P. R. Nagy, D. Mester, Z. Rolik, G. Samu, J. Csontos, J. Csóka, P. B. Szabó, L. Gyevi-Nagy, B. Hégely, I. Ladjánszki, L. Szegedy, B. Ladóczki, K. Petrov, M. Farkas, P. D. Mezei, Á. Ganyecz, *J. Chem. Phys.* **2020**, *152*, 074107.

[2] (a) A. D. Becke, *Physical Review A* **1988**, *38*, 3098-3100; (b) C. Lee, W. Yang, R. G. Parr, *Physical Review B* **1988**, *37*, 785-789; (c) A. D. Becke, *J. Chem. Phys.* **1993**, *98*, 5648-5652; (d) P. J. Stephens, F. J. Devlin, C. F. Chabalowski, M. J. Frisch, *J. Phys. Chem.* **1994**, *98*, 11623–11627.

[3] (a) B. P. Prascher, D. E. Woon, K. A. Peterson, T. H. Dunning, A. K. Wilson, *Theor. Chem. Acc.* **2011**, *128*, 69-82; (b) R. A. Kendall, T. H. Dunning, R. J. Harrison, *J. Chem. Phys.* **1992**, *96*, 6796-6806.

[4] (a) D. G. Truhlar, M. S. Gordon, *Science* **1990**, *249*, 491-498; (b) C. Gonzalez, H. B. Schlegel, *J. Phys. Chem.* **1990**, *94*, 5523-5527; (c) K. Fukui, *Acc. Chem. Res.* **1981**, *14*, 363-368; (d) K. Fukui, *J. Phys. Chem.* **1970**, *74*, 4161-4163.

[5] F. Neese, E. F. Valeev, *J. Chem. Theory Comput.* **2011**, *7*, 33-43.

[6] T. Helgaker, W. Klopper, H. Koch, J. Noga, *J. Chem. Phys.* **1997**, *106*, 9639-9646.

[7] S. Guo, M. A. Watson, W. Hu, Q. Sun, G. K.-L. Chan, *J. Chem. Theory Comput.* **2016**, *12*, 1583-1591.

[8] P. Pulay, *Int. J. Quantum. Chem.* **2011**, *111*, 3273-3279.

[9] D. Hegarty, M. A. Robb, *Mol. Phys.* **1979**, *38*, 1795-1812.
